# Effects of Chickpea in Substitution of Soybean Meal on Milk Production, Blood Profile and Reproductive Response of Primiparous Buffaloes in Early Lactation

**DOI:** 10.3390/ani10030515

**Published:** 2020-03-19

**Authors:** Francesco Serrapica, Felicia Masucci, Raffaele Romano, Fabio Napolitano, Emilio Sabia, Alessandra Aiello, Antonio Di Francia

**Affiliations:** 1 Dipartimento di Agraria, Università di Napoli Federico II, Via Università 100, 80055 Portici, Italy; francesco.serrapica83@gmail.com (F.S.); raffaele.romano@unina.it (R.R.); alessandra.aiello@unina.it (A.A.); antonio.difrancia@unina.it (A.D.F.); 2 Scuola di Scienze Agrarie, Forestali, Alimentari ed Ambientali, Università degli Studi della Basilicata, Via dell’Ateneo Lucano 10, 85100 Potenza, Italy; fabio.napolitano@unibas.it; 3 Faculty of Science and Technology, Free University of Bozen-Bolzano, Piazza Università 5, 39100 Bolzano, Italy; emilio.sabia@unibz.it

**Keywords:** chickpea, protein source replacer, primiparous Mediterranean buffalo, early lactation, milk traits, metabolic and reproductive responses

## Abstract

**Simple Summary:**

Currently, the protein needs of lactating buffaloes are mainly covered by soybean derivatives produced predominantly overseas. In order to promote the use of locally produced protein sources, in this study we tested the effects of total replacement of soybean meal by using chickpea meal, a protein-rich legume well adapted to and traditionally grown in the Mediterranean area. We evaluated the effects of these two alternative protein sources on blood profile, reproductive response and milk traits in primiparous buffaloes in early lactation. Based on our findings, chickpea meal does not impair the productive and reproductive performances of primiparous dairy buffaloes. In addition, chickpeas may represent a good alternative protein source for organic farms as it is not at risk of contamination by genetically modified cultivars.

**Abstract:**

This study aimed to evaluate the effects of the use of chickpea meal in substitution of soybean meal on plasma metabolites, reproductive response, milk yield and composition and milk coagulation traits of primiparous buffaloes in early lactation. Eighteen primiparous buffaloes were blocked by age, body weight and days in milk and equally allotted to two experimental groups from 10 to 100 days of lactation. The experimental diets consisted of the same forage integrated with two different isonitrogenous and isoenergetic concentrates containing either 210 g/kg of soybean meal or 371 g/kg chickpea. The use of chickpea meal had no negative effects on dry matter intake (*p* = 0.69), body condition score (*p* = 0.33) and milk yield (*p* = 0.15). Neither milk composition nor blood metabolites were influenced by dietary treatments (*p* > 0.05), but an increment of urea concentrations in milk (*p* < 0.05) and blood plasma (*p* < 0.001) were observed in buffaloes fed chickpeas. Moreover, no effect (*p* > 0.05) of the dietary treatment was highlighted on milk coagulation traits as well as buffalo reproductive responses. We concluded that soybean meal can be replaced by chickpea meal in the diet for primiparous dairy buffaloes in the early lactation period without impairing their productive and reproductive performance.

## 1. Introduction

Water buffalo (*Bubalus bubalis* Mediterranean type) farming is a traditional dairy enterprise of specific areas located in central and southern Italy. Milk from buffaloes is mainly used to produce the fresh soft cheese “Mozzarella di Bufala Campana”, and, to a much lesser extent, other traditional *pasta filata*-type cheeses [1,2]. Traditionally, buffalo farming has been conducted under extensive conditions, but in recent decades it underwent a rapid process of intensification due to the growing mozzarella cheese demand and to increased competition for land with other agricultural activities, chiefly horticulture [3,4]. This intensification resulted in an increment of herd sizes and in greater needs for feed produced off-farm, thus affecting the eco-efficiency and economic returns of farms [5,6,7]. Currently, the forage crop systems in buffalo farming are based almost exclusively on sorghum or maize in summer, and forage grass in winter, whereas the dietary protein are purchased off-farm, primarily as genetically modified soybean derivatives imported from countries outside the European Union [8,9]. This feed supply system, prompted by the low cost of soybean meal, has an impact on the environmental sustainability of buffalo farming, exposes farmers to market price volatility of concentrates and, at the same time, prevents the growth of the organic buffalo farming, due to the ban of solvent-extracted meals as well as genetically modified feedstuffs in this system [10,11,12]. Among the grain legumes other than soybean, chickpea (*Cicer arietinum* L.) may be a sustainable protein crop in the Mediterranean area, given the low water and nitrogen requirements, the remarkable drought-stress resistance, and the great flexibility as a rotational crop due to a long sowing window [13]. Moreover, like other legume pulse crops, chickpea can play a pivotal role in the improvement of nitrogen balance at farm-scale thanks to the symbiotic nitrogen fixation. Therefore, although mainly produced for human consumption, chickpea can represent an alternative protein and energy feedstuff especially for organic livestock farming [14,15]. A constraint upon the use of chickpea in lactating ruminants is the high percentage of rumen-degradable protein [16,17]. During early lactation, a dietary level of rumen-degradable protein exceeding the amount required from rumen bacteria, along with a negative energy balance (EB), can affect fertility including the length of postpartum anestrus, especially in primiparous cows wherein additional growth requirements can influence the severity of EB [18,19]. The few studies available on the inclusion of chickpea in the diet for lactating ruminants were largely confined to measurements of milk production [20,21,22]. Therefore, this trial was designed to examine the effects of the total replacement of soybean meal by using chickpea meal (a locally produced protein source) on milk traits, metabolic profile and reproductive performances of primiparous dairy buffalo cows in early lactation.

## 2. Materials and Methods

### 2.1. Animals, Feeding, and Management

Chickpea-belonging cultivar Sultano (Kabuli types) was organically produced in Campania, a region of southern Italy (grain yield 2.6 ± 0.13 t/ha), and was used to perform a feeding trial in a nearby buffalo dairy farm. Eighteen primiparous buffaloes were blocked by expected date of calving, age (on average, 31.5 ± 1.6 months), body weight (BW, 555 ± 40 kg) and body condition score (BCS, 6.25 ± 0.38) determined on a 1–9 scale modified for buffaloes [23], and equally assigned to one of the two dietary groups (soybean and chickpea) from 10 ± 4 days of lactation onwards. The experimental period was from March to June and covered the first 100 d of days in milk (DIM). The groups were housed in two free-stall barns with access to outdoor paddocks and equipped with stainless steel water bowls and a concrete trough. A bull of proven fertility was kept in each barn throughout the trial.

The groups were fed once a day (07:30 h) the same total mixed ration (TMR) based on corn silage, alfalfa hay and ryegrass haylage, that was integrated with two isonitrogenous and isoenergetic concentrate mixtures containing either soybean meal (210 g/kg as fed) or chickpea meal (371 g/kg as fed), in addition to the other ingredients (Table 1 and Table 2). Compared to soybean meal, the chickpea meal showed higher contents of starch, almost absent in soybean, whereas the crude protein (CP) level was less than half and characterized by a higher proportion of the soluble fractions (i.e., soluble protein (SP) and non-protein nitrogen (NPN)). Thus, in order to keep isonitrogenous and isoenergetic the two concentrates, soybean meal was substituted by chickpeas until the isoenergeic threshold and then corn gluten meal was increased to make isonitrogenous the two concentrates. Overall, chickpea and corn gluten meal contribute almost equally to the protein content of the chickpea concentrate (338 vs. 332 g/kg of CP, respectively). Batches of the two concentrate mixtures were prepared on the farm at weekly interval through a paddle mixer (Sgariboldi S.r.l., Codogno, LO, Italy). Daily, the TMR was delivered on the cement floor in front of each feed bunk, mixed by a pitchfork with the concentrate mixture according to the treatment, and then offered at 110% of the expected intake. The rations were re-approached several times daily to allow for ad libitum consumption. Cows were milked twice daily (05:00 h and at 16:00 h) in an auto-tandem milking parlor. Cows were managed in compliance with the European requirements concerning the protection of animals used for scientific purposes (Dir. 2010/63/UE) as implemented by the Italian legislation (DL n. 26, 4 March 2014).

### 2.2. Measurement, Sampling Procedure and Analytical Methods

#### 2.2.1. Animals, Feeds and Diets

The cows were weighed at the beginning and at the end of experimental period, just before the TMR morning delivery. Simultaneously, cows were BCS scored by the same trained observer blind to the treatments, using the aforementioned 9-point scale (1 = thin to 9 = obese) divided into 0.25 point scores. Changes in BW and BCS were calculated for each buffalo as the difference between the values of BW and BCS recorded at the end and the beginning of the trial. Pregnancy was determined through rectal palpation at 30-day intervals. Feed intake was weekly measured on a group basis by the difference between TMR offered and orts. The feeds and the TMR were weekly sampled, dried in a forced-air oven (at 65 °C until the constant weight), ground to pass to 1-mm screen and, then, pooled across the sampling days and by group for the chemical analyses. The procedures of the Association of Official Analytical Chemists [24] were used to determine the content of dry matter (DM; method 930.15), ash (method 942.05), crude protein (CP; method 976.05), and ether extract (EE; method 954.02) of feeds and diets. Organic matter (OM) was calculated as the difference between DM and ash content, with ash determined by combustion at 550 °C overnight. The content of neutral detergent fiber (NDF) and acid detergent fiber (ADF) were determined according to the methods of Van Soest et al. [25] using an Ankom^220^ Fibre Analyzer unit (Ankom Technology Corporation, Fairport, NY, USA). Heat-stable α-amylase (activity 17.400 Liquefon units/mL, Ankom Technology) and sodium sulphite were used in the NDF procedure and both fiber fractions were expressed as inclusive of residual ash. Acid detergent lignin (ADL) was determined by treating with 72% sulphuric acid the ADF residue [26]. Starch was determined according to the Ewers’ method as described by the standard ISO 649 [27] by using a Polax-2L polarimeter (Atago Co., Ltd., Tokyo, Japan) in 200 mm long observation tubes. Soluble protein (SP) and non-protein nitrogen (NPN) were determined according to the procedure of Licitra et al. [28]. Each analysis was performed at least in duplicate. The non-fibrous carbohydrates (NFC) of feeds and diets was calculated as 100 − (%NDF + %CP + %EE + %Ash) [29]. Net Energy for Lactation (NE_L_) content was estimated according to Sauvant and Nozière [30]. The nutrient requirements, estimated according to a milk yield of 8.1 kg/d and a BW of 575 kg, were NE_L_ 6.27 MJ/kg DM, CP 13.6 %DM, NDF 48.3 %DM [31,32].

#### 2.2.2. Milk Yield and Quality

Milk yield of each cow was measured at each milking by a computerized system and sampled at 2-week intervals. Samples were collected in sterile plain jars, temporarily kept refrigerated at 4 °C and sent to laboratory to be analyzed the same day of collection for fat, protein, lactose (Milkoscan 605, Foss Electric, Hillerød, Denmark), urea (CL-10 Plus, Eurochem, Rome, Italy) and somatic cell count (SCC; Fossomatic 90, Foss Electric, Hillerød, Denmark). Every three weeks, bulk milk of each group was collected to determine the fatty acids (FA) composition of milk fat and clotting ability. Milk fat extraction was performed by using the Röse-Gottlieb method. The Supelco 37 Component mix (Supelco, Bellefonte, PA, USA) and a mixture of conjugated linoleic acid isomers (Nu-Chek Prep. Inc. Elysian, MN, USA) were used as external standards for the gas chromatography analysis. Values < 0.1 were not quantified. Further details are given elsewhere [33,34]. Milk clotting ability, expressed as rennet clotting time (r), curd-firming time (K_20_) and curd firmness (A_30_), was evaluated using a thromboelastograph (Formagraph, Foss Electric, Hillerød, Denmark) at the technical time of 30 min by adding to 10 mL of milk, at 35 °C, 0.2 mL of a diluted solution (1.6:100) of liquid rennet (1:15,000 rennet unit; 90% chymosin; Chr. Hansen, Parma, Italy) [35]. The value of milk SCC was log transformed to base 10. Based on milk composition, the buffalo standard milk (fat- and protein-corrected milk, FPCM, at 8.3 and 4.73% of fat and protein, respectively) was calculated as reported by Bovera et al. [36], whereas Mozzarella cheese yield was estimated by using the equation of Altiero et al. [37]:

(1) $$\mathrm{Mozzarella}\;\mathrm{cheese}\;\mathrm{yield}\;\left(\%\right)=\left[3.5\times \left(\mathrm{milk}\;\mathrm{protein},\;\%\right)+1.23\times \left(\mathrm{milk}\;\mathrm{fat},\;\%\right)\right]-0.88$$

#### 2.2.3. Blood Metabolic Profile and Reproduction Response

Cows’ blood samples were individually collected every two weeks in the morning, before the daily ration delivery, via jugular venipuncture, using 10 mL Li-heparinized vacuum tubes (BD Vacutainer, Becton Dickinson Italia S.p.a., Milano, Italy). The samples were temporarily placed in a thermo-isolated cooled box at 4 °C and centrifuged within 30 min in the field at 3000 rpm for 10 min (Hettich EBA 270, Tuttlingen, Germany). Haemolysed samples were excluded. Recovered plasma was split into aliquots and, then, frozen on dry ice to permit safe transport to the laboratory, wherein they were kept at − 20 °C until analyzed. Plasma samples were assayed using commercial kits (Futura System S.r.l., Roma, Italy) and a spectrophotometer (Jasco V-530, Jasco, Tokyo, Japan) for total protein (TP), albumin, calcium, phosphorus, triglycerides (TGR), total cholesterol (CHC), glucose (GLU), non-esterified fatty acids (NEFA), blood urea (BU), and ß-OH-butyrate (BHBA). Alanine aminotransferase (ALT) and aspartate aminotransferase (AST) were assayed using commercial kits (bioMérieux; Marcy-l’Etoile, France) following the producer’s suggested procedures. Blood globulin content was obtained as the difference between total protein and albumin. For the cows that became pregnant during the experimental period, the days open, calving interval and the conception rate were calculated. The days open are the number of days from calving to date of conception determined by pregnancy diagnosis, the calving interval is given by the sum of standard gestation length (308 days) and the number of days open [38]. Conception rate was calculated on a group basis as the number of cows confirmed pregnant divided by the total number of cows (*n* = 12) [39].

### 2.3. Statistical Analyses

Before statistical analysis (SAS statistical software, 1990), normality and homogeneity of variance of data were tested by the Shapiro–Wilks and Levene tests, respectively. Milk production and blood parameters underwent analysis of variance for repeated measures (mixed procedure) with the dietary treatment (soybean and chickpea) as a non-repeated factor and sampling time and diet × sampling time as repeated factors. The cow variance was considered as random and utilized as the error term to test the main effect of the diet. Dry matter intake, milk coagulation properties, and milk FA composition were analyzed by one-way analysis of variance (General Linear Model procedure) to determine the fixed effects of the dietary treatment (soybean and chickpea). Statistical significance was declared at *p* < 0.05 and tendencies discussed at *p* < 0.10.

## 3. Results and Discussion

### 3.1. Feeds, Dry Matter Intake, Body Weight (BW) and Body Condition Score (BCS)

The chemical composition of the protein feeds, concentrate mixtures and experimental diets are given in Table 2.

Chickpea composition was consistent with previous reports for the same cultivar and for other chickpea Kabuli biotypes produced in different cultivation areas [16,17,40,41]. Despite the differences between the two protein feeds, the corresponding concentrate mixtures showed comparable protein contents, but the SP fraction remained higher (+ 47.3 g/kg DM) in the chickpea concentrate and, as a consequence, in the diet (+ 18.0 g/kg DM). Overall, the chickpea and soybean diets can be considered equivalent and able to meet the nutritional requirements for buffaloes in early lactation [31]. Dry matter intake was not influenced by the dietary treatments (Table 3), confirming the observations of previous studies on lactating cows [22], ewes [20,21], and steers [42]. Similarly, no differences were observed among the two groups for BW and BCS at the end of the trial, in agreement with other studies [20,21] (Table 3). At 100 days postpartum, both groups showed a less severe BCS variation as compared to previous data on multiparous buffaloes [32,43,44,45,46]. This observation may suggest that depletion of body reserves in buffaloes is affected by parity, whereby the primiparous buffaloes would mobilize less body fat reserves than multiparous animals during the post-partum period, as extensively reported for dairy cows [47,48,49].

### 3.2. Milk Traits and Fatty Acids Composition

Milk yield and composition of the soybean and chickpea groups are given in Table 4. The replacement of the protein source did not affect actual and FPCM milk yield, as well as the contents of fat, protein and lactose and SCC. Accordingly, no negative effects were observed among groups in terms of estimated mozzarella yield, rennet clotting time, curd-firming time and curd firmness, which are all parameters related to milk fat and protein contents [31,50]. Milk urea content was higher (*p* < 0.05) in chickpea milk, most likely due to the high solubility of chickpea protein. However, no differences were observed for milk protein content, whose levels were consistent with data reported for buffaloes at the same calving order and DIM [51,52,53].

Data on the effect of chickpea on milk production in dairy ruminants are scanty and inconsistent, and no studies have been conducted on buffaloes. In early lactation dairy cows the total replacement of soybean meal by chickpea improved milk yield and milk fat, while milk protein content decreased [22]. This latter result may be due to excess soluble protein that may have reduced microbial protein synthesis thus lowering milk protein content [54]. Indeed, an unbalanced intake of fast degradable protein can overcome the capacity of rumen bacteria to capture ammonia nitrogen, originated from protein degradation, for microbial growth, thus reducing the intestinal protein supply and, as a consequence, the milk protein content [29,55,56]. Conversely, and in accordance with our results, Christodoulou et al. [21] and Bonanno et al. [20] did not observe any effect of partial or total replacement of soybean with chickpea on ewes’ milk yield and quality. The lack of productive responses we observed may indirectly indicate a balanced protein and energy supply of the chickpea diet, since, unlike dairy cows, buffaloes fed unbalanced diet tend to drop milk yield and quality rather than mobilize nutrients from body reserves [57]. In addition, according to Campanile et al. [46] milk yield response of buffaloes to higher rumen undegradable protein levels (as is the case of soybean diet in this trial) can be observed only if CP intake is barely enough to meet animal requirements and, in any case, would be lower as compared to dairy cows. The milk FA composition of chickpea and soybean groups is in Table 5. The dietary protein replacement had no effects on FA profile, apart from a tendency (p = 0.06) for linoleic acid to be higher in the chickpea group, probably related to a high content of C18:2 in chickpea, as reported by Bonanno et al. [20,58].

The feeding strategies able to modify the FA composition of milk fat are mainly based on the use of pasture/fresh forage, oils and oil-rich feeds [59,60,61]. However, in this trial, no fresh forage was used and the fat percentages of the two diets were very close and, thereby, the effect of diet was not observed. Similar results were also reported by Bonanno et al. [20]. The FA profile of the two groups was comparable to those displayed by pluriparous buffaloes at different stages of lactation [62,63,64], thus indicating that parity and time from delivery may have a negligible effect on FA, as also observed in dairy cows [65].

### 3.3. Metabolic Profile and Reproduction Responses

The metabolic profile and reproduction performances of the chickpea and soybean groups are summarized in Table 6. The use of chickpea meal had no negative effects on plasma metabolites, with values within the normal range for dairy buffaloes [66,67]. This result is of importance because it was obtained in animals particularly prone to metabolic disorders, such as primiparous cows in early lactation. Nevertheless, in agreement with the results on milk urea content, the chickpea group showed a blood urea concentration higher than the soybean group (*p* < 0.001).

As suggested by Marino et al. [68], the haematochemical variables may contribute to giving useful indications about the nutritional status of the animals. In ruminants, plasma urea mainly derives from the conversion of ammonia produced in the rumen and from amino acids deaminated by the liver [69]. As a consequence, excesses of dietary protein, of readily degradable protein, as well as an unbalanced protein–energy ratio or the lack of limiting amino acids for rumen microflora, can overtake the microbial synthesis capacity and lead to an increment of ammonia escaping from the rumen and, subsequently, of plasma urea [56,70]. Thus, overall, plasma urea represents a nitrogen metabolism indicator, whose changes reflect dietary CP content, the composition of ingested amino acids, and the rate and extent of protein and carbohydrate degradation in the rumen [71]. Since the diets were formulated to be isonitrogenous, the increase of blood urea concentration observed in the chickpea group may be likely due to the different proportions of protein-degradable fractions. High blood urea concentrations have been associated with reduced reproductive efficiency in dairy cows, with an increase of the number of days not pregnant and a lower fertility rate [72]. However, in our study, days open and conception rates were not different across the treatments (Table 6). Therefore, in agreement with Campanile et al. [46], increased blood urea concentration seems not to influence the reproductive efficiency of naturally mated dairy buffaloes. The same authors observed that the dietary excess of ruminal degradable protein increased blood urea without affecting blood ammonia level; at the same time, no changes of both metabolites in the vaginal mucus occurred. The reasons for these responses, markedly in contrast with what is usually observed in dairy cows, may be due to the better use of nitrogen in the buffalo, given the more favorable intra-ruminal environment for NPN-using bacteria, and the higher liver efficiency in detoxifying blood ammonia into urea [45,73,74]. Moreover, independently from blood urea levels, a lower ammonia diffusion in the buffalo uterus has been hypothesized compared to cattle, which would contribute to mitigating the detrimental effects of ammonia on embryonic development [46]. However, a negative correlation between blood urea and buffaloes’ reproductive efficiency has been highlighted in other studies [23,75,76]. The lack of the effects observed herein could be also attributed to the interference of reproductive seasonality, since the increase in daylight hours tends to penalize fertility in female buffaloes, suppressing or masking any potential relationship with dietary treatments [23]. Such responses can be particularly marked in primiparous buffaloes that tend to delay the post-partum cyclicity resumption and to have a low ovulation rate during early lactation, also in relation to the concomitant growth requirements [77,78]. Accordingly, both groups showed a higher number of days open and lower conception rates compared to naturally mated multiparous buffalo cows at the same calving distance [76,79]. Similarly, the conception rate observed in this study was lower than that reported for primiparous buffaloes in late lactation [80].

## 4. Conclusions

The use of chickpea in the diet for primiparous buffaloes increased urea content in blood and milk, but did not affect BW, BCS, milk yield and composition. In addition, no negative effects were observed on reproductive performances. However, this latter aspect should be explored in more detail, since the reproductive seasonality of buffaloes may have masked the effect of diet. Overall, despite the high content of soluble protein, no detrimental effects related to the use of chickpea was observed. This result is of importance since it has been obtained in animals prone to metabolic disorders, such as primiparous cows in early lactation. Therefore, we conclude that chickpea appears a promising protein feed for conventional buffalo farms, as a crop well adapted to the local Mediterranean conditions, and a good alternative protein source for organic farms as it is not at risk of contamination by genetically modified cultivars.

## Figures and Tables

**Table 1:** Chickpea and soybean meal nutritive value characteristics (% of dry matter, DM, if not otherwise stated).

Item	Protein Sources	
	Chickpea Meal	Soybean Meal
DM (% as fed)	89.3	88.0
Ash	3.3	6.8
Crude protein	21.9	48.4
Ether extract	4.0	2.0
NDF	27.7	15.34
ADF	4.8	10.91
ADL	1.2	1.1
NFC	49.6	27.5
Starch	48.9	3.7
SP (% CP)	54.0	5.9
NPN (% CP)	27.3	11.0
NE_L_ (MJ/kg DM)	8.64	8.14

DM, dry matter; CP, crude protein; NDF, neutral detergent fiber; ADF, acid detergent fiber; ADL, acid detergent lignin; NFC, non-fibrous carbohydrate; SP, soluble protein; NPN, non-protein nitrogen; NE_L_, net energy of lactation.

**Table 2:** Ingredients and chemical composition (% of dry matter, DM, if not otherwise stated) of the experimental concentrates mixture and total mixed rations.

Item	Concentrate Mixture		Total Mixed Ration	
	Soybean	Chickpea	Soybean	Chickpea
Ingredients ^1^				
Corn meal	403.2	161.3	-	-
Soybean meal	209.7	-	-	-
Chickpea meal	-	371.0	-	-
Wheat bran	161.3	161.3	-	-
Wheat flour middlings	145.2	161.3	-	-
Corn gluten meal	64.5	129.0	-	-
Vitamin and mineral premix^2^	16.1	16.1	-	-
Corn silage	-	-	15.0	15.0
Reygrass hay	-	-	3.5	3.5
Alfalfa hay	-	-	1.5	1.5
Concentrate mixture	-	-	6.2	6.2
Chemical composition				
DM (% as-fed)	88.5	88.9	57.7	57.8
Ash	6.0	5.9	7.4	7.3
Crude protein	24.3	24.1	13.2	13.1
Ether extract	3.5	3.9	2.6	2.7
NDF	22.4	25.9	44.9	46.1
ADF	7.5	6.6	27.5	27.2
ADL	1.8	1.8	4.1	4.1
NFC	45.4	43.0	32.6	31.7
Starch	39.4	42.8	21.8	23.0
SP (% CP)	4.8	24.4	19.3	32.9
NPN (% CP)	11.5	17.9	22.3	26.6
NE_L_ (MJ/kg DM)	8.07	8.14	6.08	6.05

^1^ Expressed as g/kg as-fed for the concentrate mixture and as kg/day as-fed for the total mixed rations; ^2^ Premix containig (per kg, based on the manufacturer’ declared content): 4,000,000 IU of vitamin A; 100,000 IU of vitamin D3; 1500 mg of vitamin E; 1400 mg of vitamin B6; 1400 mg of vitamin C; 1100 mg of vitamin B1; 500 mg of vitamin B2; 5000 mg of choline chloride; 1000 mg of biotin; 800 mg of pantothenic acid; 700 mg of niacinamide; 180 g calcium; 38 g of phosphourous; 70 g of sodium; 15 g of magnesium; 1000 mg of S as copper-II-sulfate; 1600 mg of Mn as manganese-II-oxide; 5400 mg of Zn as zinc sulfate, monohydrate; 80 mg of I as calcium iodate, anhydrous; 10 mg of Se as sodium selinte. DM, dry matter; CP, crude protein; NDF, neutral detergent fiber; ADF, acid detergent fiber; ADL, acid detergent lignin; NFC, non-fibrous carbohydrate; SP, soluble protein; NPN, non-protein nitrogen; NE_L_, net energy of lactation.

**Table 3:** Dry matter intake, body weight and body condition score (Least Ssquare Means) of buffaloes over the first 100 days of lactation.

Item	Diet		SEM	*p* Value
	Soybean	Chickpea		
DMI, kg/head/day	16.0	15.9	1.28	0.69
BW (kg) 10 days postpartum	557.5	552.5	20.89	0.81
BW (kg) 100 days postpartum	575.1	571.7	19.45	0.87
BWC (kg) 10 to 100 days postpartum	17.6	19.2	3.52	0.34
BW Variation, %	3.2	3.5	0.85	0.44
BCS 10 days postpartum	6.3	6.1	0.19	0.33
BCS 100 days postpartum	7.50	7.75	0.14	0.12
BCSC 10 to 100 postpartum	1.2	1.6	0.15	0.14
BCS Variation, %	21.9	22.6	5.80	0.80

DMI, dry matter intake; BW, body weight; BWC, body weight change; BCS, body condition score; BCSC, body condition score change; SEM, standard error of mean.

**Table 4:** Effect of diets containing soybean meal and chickpea meal as main protein source on daily milk yield and composition, milk-clotting properties, and estimated mozzarella cheese yield (Least Square Means).

Item	Diets		SEM	*p* Value
	Soybean	Chickpea		
Milk yield, kg/day	8.9	8.8	0.05	0.15
FPCM, kg/day	8.8	8.7	0.05	0.24
Fat, g/kg	8.15	8.19	0.02	0.19
Protein, g/kg	4.72	4.69	0.01	0.31
Lactose, g/kg	4.87	4.89	0.09	0.20
Urea, mg/dL	38.9	39.4	0.17	0.03
SCC, lg n. cells/mL	5.9	5.8	0.14	0.50
Clotting properties				
r (min)	17.4	17.3	0.14	0.83
K_20_ (min)	3.5	3.4	0.07	0.20
A_30_ (mm)	39.3	39.1	0.33	0.39
Mozzarella cheese yield ^1^ (%)	25.7	25.6	0.06	0.79

^1^ Calculated as: [3.5 × (milk protein, %) + 1.23 × (milk fat, %)] − 0.88; fat- and protein-corrected milk (FPCM), milk corrected to 8.3% of fat and 4.73% of protein; SCC, somatic cells count; r, rennet clotting time; K_20_ curd-firming time; A_30_,curd firmness; SEM, standard error of mean.

**Table 5:** Fatty acids composition (% of total FA) of milk (Least Squre Means) produced by buffaloes fed soybean meal and cickpea meal as main protein sources.

Item	Diets		SEM	*p* Value
	Soybean	Chickpea		
C4:0	3.7	3.5	0.25	0.33
C6:0	1.7	2.0	0.45	0.15
C8:0	0.9	1.0	0.24	0.57
C10:0	1.99	1.91	0.37	0.69
C12:0	2.8	2.4	0.53	0.29
C14:0	11.4	11.0	1.02	0.58
C14:1*cis*-9	1.3	1.2	0.16	0.47
C14:1*trans*-9	0.36	0.36	0.04	0.94
C15:0	1.2	1.2	0.13	0.56
C16:0	32.3	30.7	2.31	0.25
C16: 1*trans*-9	0.39	0.43	0.09	0.51
C16: 1*cis*-9	1.8	1.8	0.40	0.84
C17:0	0.5	0.60	0.07	0.14
C18:0	11.9	12.6	1.97	0.53
C18:1*trans*-11	2.1	2.2	0.62	0.80
C18:1cis-9	21.9	22.9	2.09	0.43
C18:2 n-6 cis	1.9	2.1	0.14	0.06
C20:0	0.21	0.23	0.03	0.39
C18:3 n-3	1.0	1.0	0.25	0.50
C18:2 *cis*-9, *trans*-11	0.62	0.51	0.16	0.29
Short-chain	8.2	8.4	1.14	0.69
Medium-chain	51.6	49.3	3.48	0.27
Long-chain	40.2	42.3	3.92	0.38
Saturated	68.5	67.3	2.28	0.39
MUFA	28.6	29.5	2.32	0.51
PUFA	2.9	3.2	0.34	0.20

FA, fatty acids; MUFA, monounsaturated fatty acids; PUFA, polyunsaturated fatty acids; SEM, standard error of mean.

**Table 6:** Blood metabolic profile and reproduction response (Least Square Means) of buffaloes fed soybean meal and cickpeas meal as main protein sources.

Item	Diets		SEM	*p* Value
	Soybean	Chickpea		
Blood metabolic profile				
TP, g/L	76.1	75.9	0.95	0.89
Globulin, g/L	42.0	41.8	0.76	0.81
Albumin, g/L	34.1	34.2	0.65	0.92
BU, mmol/L	11.7	12.6	0.12	<0.001
GLU, mmol/L	4.2	4.3	0.10	0.25
CHC, mmol/L	2.3	2.3	0.07	0.73
NEFA, mmol/L	0.42	0.41	0.01	0.65
BHBA, mmol/L	0.28	0.30	0.01	0.39
TRG, mmol/L	0.14	0.13	0.005	0.12
Calcium, mmol/L	2.5	2.5	0.04	0.49
Phosphorous, mmol/L	1.8	1.8	0.04	0.56
ALT, IU/L	54.5	55.0	0.66	0.54
AST, IU/L	161.5	162.9	2.04	0.64
Reproduction response				
Days open, day	71.8	73.5	13.57	0.85
Calving interval, day	379.8	381.5	13.41	0.23
Concepition rate ^1^, %	55.6	44.4	-	-

^1^ Calculates on a group basis as the number of cows confirmed pregnant divided by the total number of cows × 100; TP, total protein; BU, blood urea GLU, glucose; CHC, total cholesterol; NEFA, non-esterified fatty acids; BHBA, ß-OH-butyrate; TRG, triglycerides; ALT, alanine aminotransferase; AST, aspartate aminotransferase; SEM, standard error of mean.
